# On the Sulphate of Iron

**Published:** 1860-06

**Authors:** R. C. Hamill


ARTICLE 2.

### ON THE SULPHATE OF QUININE.

READ BEFORE THE CHICAGO ACADEMY OF MEDICAL SCIENCES, APRIL 7TH, 1860,

BY THE PRESIDENT, DR. R. C. HAMILL.

In a paper which I read before this society, at its February meeting, I advanced the opinion that we were indebted to the action of the sulphate of quinine for a certain febrile condition, that puts on many of the phenomena of typhoid fever; and which, not unfrequently, has been styled and treated as typhoid, especially by that class of doctors who let no opportunity slip to give undue gravity and consequence to all forms of disease that fall under their care.

I gave as my belief, that this irritative form of fever was brought about by the exhibition of quinine at stages of disease when the system was not prepared for its healthful action; viz., that the secretory system of the alimentary canal was acting imperfectly—the amount secreted small and vicious—that the alkaline properties of those secretions which furnish solvents for uric acid and urea were deficient—and that by administering a drug which arrested the elimination of uric acid by the kidney, (which effect, according to Dr. Ranke, is produced by the sulphate of quinine,) we contravene a sanative law of the organism, and throw the burthen from the kidney upon the stomach and bowels, giving rise to copious, very fluid, brown, intestinal evacuations, dry tongue, stupor, delirium, &c.—and that material, not appropriated for the repair of tissue, was retained in the sysiem.

It will readily be admitted that there is an improper as well as a proper time for the exhibition of every drug or medicinal agent, that has any power or curative virtue entitling it to a place in the materia medica; and the application of this principle obtains in relation to quinine as effectually as it does to any other drug that exalts or depresses vital action.

A small dose of opium, given in the morning, may develop phrenitis; if delayed until noon, may produce the most delightful and refreshing quiet—and if deferred until night, may cause a profound stupor from which the patient will never awake. Quinine is not a remedy of such immediate consequences, but it is an agent of acknowledged potency, or it would never have found its advocates in the treatment of almost every disease that preys on human organism.

I stated in that paper, that in my observation, good results had not followed its exhibition where the tongue was dry, and cutaneous elimination was arrested. And I do not remember of witnessing untoward effects where the tongue and cutaneous surface were moist. Every physician who has practiced medicine in a malarious district of country, knows that in the apyrexia of intermittant fever he can administer 15 or 20 grs. of quinine with almost a certainty of arresting the disease, and that in periodic disease of almost every kind he can rely upon it with the greatest confidence.

During the intermission of the paroxysm of fever, the skin is bathed in perspiration and the tongue is moist; if you increase the peristaltic action of the bowels, you have an easy victory. In the periodic forms of neuralgia, the success is equally sure if the tongue and skin are in that state which gives evidence of secretion and elimination. It undoubtedly has, so far as disease is periodical, a restorative power, and when given six or eight hours in advance of the paroxysm, accomplishes its mission. But suppose the certain knowledge we have, be disregarded, and the quinine be given a half hour or hour before the expected chill or fever, and what is the effect? Certainly not an arrest of the paroxysm, but a more violent attack of the fever. The exaltation produced by the quinine is superadded, and in all probability the patient will exhibit the frenzy of brain disease—he will, unquestionably, complain of a most intolerable, bursting head ache, and if you have an intermission, it will be after a prolonged exacerbation. A continuation of the medicine will arrest the next paroxysm, but you will find that recovery is not perfect, that the organism does not rally to its normal state with its usual elasticity. It will be found, occasionally, under such circumstances, that the fever does not subside, but assumes a low and continued type, such as I have before alluded to.

I give my own experience and observation, and I think a careful observer will find facts enough to lead him to the same conclusions, in any malarial district of country. I would not choose him for a careful observer, who has settled down upon the theory, that quinine is anti-malarious, and therefore applicable to all cases of malarial origin. I believe that the great cardinal virtue of quinine is its anti-periodic property, which is its known power. I believe that it is a stimulant of its own kind, and that any tonic property which may be attributed to it, is the result of a stimulant to the nerves and absorbents of the stomach, thereby aiding the powers of assimilation and nutrition.

A supposed action upon the liver has grown out of the fact, that hepatic derangement so very generally accompanies the fevers of malarial districts, and to which derangements intermittents formerly were attributed.

There is scarcely a functional obliquity that has not in the hand of some one found a remedy in this drug, which fact is positive proof of the wide spread ignorance of its properties, as well as of the resorts to which some doctors are driven when they really do not know what to do, yet feel that something must be done, or they will suffer either in reputation, or in the loss of practice. What a great amount of physic could be saved, and probably lives too, if the physician was obliged to furnish an intelligent reason for every dose he prescribes.

Some of the advocates of a quinine treatment of fever, claim that it enters the circulation, and by supplying a deficiency in the blood, arrests the degradation of the tissue and thus breaks up a fever. The examination of this view leads entirely into the fields of speculation. Inviting though it be, I will not enter it farther than simply to notice one or two of the reasons for the hypothesis.

The main argument to sustain this view, is based upon a fancied resemblance between quinine and one of the ingredients of healthy blood, which is styled *Taurine*, and furnished by the liver. The similarity of the two substances is founded upon the resemblance which they bear to each other in the following particulars. That is, both are tonic and crystalizable, so far identical; each contains carbon, hydrogen, oxygen and nitrogen, but in very diverse proportions; and taurine has two parts of sulphur which quinine has not.

Taurine, C 4, H 7, O 1, N 1 and S 2.

Quinia, C 20, H 12, O 2, N 2.

Uric acid, C 10, H 4, O 6, N 4.

Urea, C 2, O 2, N 2, H 4.

I deny the tonic property of quinine, as taken for granted by Dr. Headland and those who adopt his views, and find no argument in the discussion of this point which would not apply with as great, if not greater force, to its exclusion, upon the principle that it has so many points of resemblance to urea and uric acid, which are poisons, and their retention in the blood is the cause of irritative fever. Quinine, urea, and uric acid, all have the same four elementary constituents, and in proportions relatively much nearer than quinine and taurine, and are equally soluble and crystalizable—moreover, taurine has never been discovered in the blood.

Upon the supposition, that that view is correct, viz., that it enters the blood and is accepted as a substitute for the supposed missing ingredient in intermittent fever, I cannot divine what justification or authority any one can deduce for the indiscriminate use of the drug in the medication of fevers of malarious origin.

You have increased degradation of tissue, with exalted action in remittent fever, and you administer a medicinal agent that you call a stimulant, tonic and sedative. Its first action is stimulant. Now I should look for its effects as a stimulus first, and should expect to acquire moral power over an enraged mob of combatants in the heat of a melee by administering stimulants to them—to soothe and quiet their unbridled passions by liberal potations of brandy, as readily as I could expect to overrule a fever by the exhibition of a stimulant tonic during the exacerbation. The heat and passion of the mob may be overruled by partaking of the stimulus until the powers of life are debauched into beastly intoxication, and by some such analogous action a fever may be conquered, but I leave it for those, to accept and bear the consequences of such medication, who adopt the practice.

The undue proportion of super annuated constitutions, suffering from dropsical effusion, from rheumatism, from paralysis, enlarged spleens and indurated livers, as well as the greater prevalence of low types of fever, suggest the question whether they are all chargable to the direct influence of that subtle, unknown poison which we are taught to call malaria. I yield to no one in my admiration of the sulphate of quinine in the treatment of disease where it has a known virtue; but I must be convinced that the system is in that state of preparation which admits of the development of its legitimate power, before I am willing to exhibit it even in a case of ague. It is an established maxim that that which is powerful for good when rightly directed, is equally effective for evil, when pointed in the wrong direction. This principle has a peculiar fitness in its application, to the effect of drugs upon the human organism; and the opinion, that if it does no good, it can do no harm, is not to be entertained in relation to quinine any more than to morphine, strychnine, or arsenic. Its immediate injurious constitutional effects may not be cognizable in the present state of our medical knowledge, but when, in the course of time, science shall unveil its properties and powers, a more circumspect use of it will be inaugurated.

But to return to the position that it enters the circulation, and exhibits its power there. Give a patient, in fever, twenty or forty grains of quinine. It is admitted into the circulation and carried through all its ramifications, accompanying, perhaps impregnating the elements of tissue until they are degraded into effete material and seek an exit from the organism. Where is it to go? The portals of the skin are all closed, the kidneys and the alimentary canal are the only other resorts. We find that after the quinine has been taken, that one of the solids of the urine has diminished very materially, which ought to be a matter of congratulation, as giving evidence that the destructive process is less active, there is no waste passing off by the skin, and if there is no increase by the bowels we will be at a loss to know what is the reason—the fever is still as persistent and the destructive tendencies equally imminent—where is the disintegrated tissue? Suppose that the process of degradation or the metamorphosis of tissue has not reached the condition that will admit of its exit through the kidneys, consequent to the action of the quinine. But it may, by the time it reaches the stomach and bowels, be in a state that will permit of its escape in that direction, and thus we have the brown, copious, watery evacuations. Again, it may not find egress from any point and be retained in the system. I know that analysis has not detected these partly degraded elements of tissue in the blood, yet there are arguments which render such a conclusion altogether reasonable. For instance, elimination in fever is, in a large proportion of cases, not correspondent to the amount of food and fluids taken into the stomach. I have frequenly been asked by inquisitive old ladies, "what becomes of all the water the patient, whose tongue is parched with a burning fever, drinks?"

Upon what other hypothesis can we account for the large critical discharges that accompany the sudden lowering of temperature and recession of fever, sometimes by the bowels, but more frequently by the skin and kidneys. And, again, the secondary forms of disease that follow the exanthemata, and other forms of fever, appear to favor of a retained pernicious material in the blood which seeks this method of expressing itself.

